# Systemic inflammatory syndrome in children during COVID-19 pandemic in Ceará state, northeastern Brazil: an observational study

**DOI:** 10.1590/0037-8682-0383-2021

**Published:** 2021-11-12

**Authors:** Luís Arthur Brasil Gadelha Farias, Magda Moura de Almeida, Pâmela Maria Costa Linhares, Bruno Cavalcante Fales de Brito, Roberto Jorge Colares Duarte, Roberio Dias Leite, Marco Aurelio Palazzi Safadi, Luciano Pamplona de Góes Cavalcanti

**Affiliations:** 1 Hospital São José de Doenças Infecciosas, Fortaleza, CE, Brasil.; 2 Secretaria de Saúde do Estado do Ceará, Fortaleza, CE, Brasil.; 3 Universidade Federal do Ceará, Faculdade de Medicina, Departamento de Saúde Comunitária, Fortaleza, CE, Brasil.; 4 Centro Universitário Christus, Faculdade de Medicina, Fortaleza, CE, Brasil.; 5 Santa Casa de São Paulo, Faculdade de Ciências Médicas, Departamento de Pediatria, São Paulo, SP, Brasil.

**Keywords:** COVID-19, SARS-CoV-2, Multisystem inflammatory syndrome in children, Children

## Abstract

In this study, we report the occurrence of multisystemic inflammatory syndrome among 64 children (2 deaths) with recent severe acute respiratory syndrome-related coronavirus 2 (SARS-COV-2) infections in the northeast region of Brazil. The major clinical symptoms and signs reported were exanthema (60.9%), abdominal pain (56.3%), nausea and vomiting (46.9%), diarrhea (37.5%), and dyspnea (37.5%). Laboratory findings revealed that the levels of C-reactive protein (75.0%), hemoglobin (51.6%), D-dimer (48.4%), lymphocytes (43.8%), LDH (45.3%), AST (42.2%), ALT (51.6%), and ferritin (48.4%) were above the reference values for a given age and gender. The clinical findings were similar to those observed in Kawasaki disease, although it represents a separate entity, emphasizing the need for proactive surveillance and early treatment.

Since the emergence of the Severe Acute Respiratory Syndrome Coronavirus 2 (*SARS-CoV-2*) in China, in December 2019, the coronavirus disease 2019 (COVID-19) has affected more than 106 million people around the world^1^. Brazil was the most affected country in Latin America, with approximately 9.5 million cases and 230,000 SARS-CoV-2-associated deaths as of February 2021^2^. COVID-19 was declared a pandemic by the World Health Organization on March 11 2020, representing the most challenging public health crisis faced by this generation^2^.

A striking feature of the COVID-19 pandemic is that relatively lower number of cases have been reported in children and adolescents as compared to adults^3^. Although most children and adolescents infected with SARS-CoV-2 are asymptomatic or present mild symptoms, in rare cases, children can be affected, and clinical manifestations may differ from those of adults. A rare, potentially severe case of multisystem inflammatory syndrome in children (MIS-C) was initially reported in the UK and then globally to occur days to weeks following the acute SARS-CoV-2 infection. MIS-C represents a growing concern in the pediatric population^4^. The clinical characteristics of MIS-C share similar features with that of Kawasaki disease (KD), KD shock syndrome, macrophage activation syndrome (MAS), and toxic shock syndrome (TSS)^4^ ^,^ ^5^.

The state of Ceará in the northeast region was one of the most affected places in Brazil, with incidence rates of COVID-19 to be as high as 13,303/100,000 hab as of December 2020^6^.

To further understand the epidemiological and clinical patterns of this rare syndrome in low- and middle-income countries, we provide in-detail description of all cases of MIS-C temporally associated with COVID-19 reported between May 1 and December 31, 2020, in the State of Ceará. Figure 1 shows the number and temporal distribution of confirmed COVID-19 cases and deaths as of the epidemiological week of symptom onset in the state of Ceará, Brazil, 2020-2021.

**FIGURE 1:**
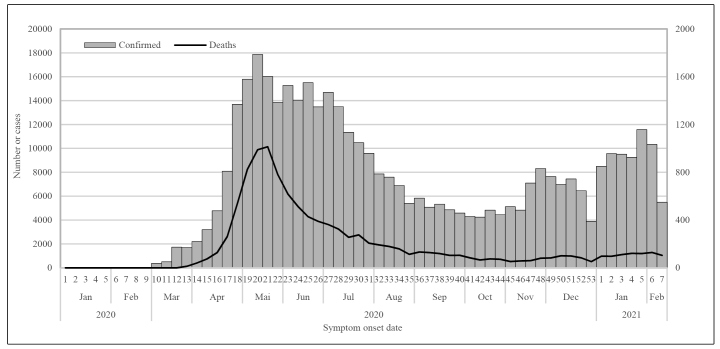
Number and temporal distribution of confirmed COVID-19 cases and deaths, by the epidemiological week of symptom onset. Ceará, Brazil, 2020-2021

We followed the case definition of MIS-C proposed by the Ministry of Health of Brazil, PAHO, and WHO,^7^ following the RECORD guidelines.

Hospitalized or fatal cases with: (1) Fever and elevated inflammatory markers within 3 days or more; (2) at least two of the following symptoms: i) rash or bilateral non-purulent conjunctivitis or mucocutaneous inflammation signs (oral, hands, or feet); ii) hypotension or shock; iii) features of myocardial dysfunction, pericarditis, valvulitis, or coronary abnormalities (including echocardiogram findings or elevated troponin or N-terminal pro B-type natriuretic peptide); iv) evidence of coagulopathy (elevated prothrombin time, partial thromboplastin time, and elevated D-dimers); and v) acute gastrointestinal problems (diarrhea, vomiting, or abdominal pain); (3) Elevated inflammatory markers such as C- reactive protein (PCR), Erythrocyte Sedimentation Rate (ESR), Lactate Dehydrogenase (LDH), ferritin, and procalcitonin; (4) Excluded cases with other microbial cause of inflammation; and (5) Positive RT-PCR, antigen test, or serology; or any contact with patients with COVID-19^7^.

A total of 64 cases were included in the study, with 2 reported deaths (case-fatality rate of 3.1%). Most cases occurred in the month of July 2020 (n =31; 48.4%). About 48% of the patients were male. The median age was 7 years (range,0-16 years). The cases occurred in 17 different municipalities, predominantly in Fortaleza, the capital of Ceará (42; 65.6%). These cases were reported by five hospitals, particularly from the reference hospital in the State, Albert Sabin Hospital (N = 41; 64.1%). The majority of cases had positive testing, either real-time reverse transcription polymerase chain reaction (RT-PCR) and/or antigen testing, from nasopharyngeal specimens and less frequently serologic testing for SARS-CoV-2 (Table 1).

**TABLE 1:** Demographic, clinical and laboratory diagnosis features of 64 children and adolescents with MIS-C temporally associated with SARS-CoV-2 infection from Ceará, Brazil.

Variable	N = 64
Age, years, median (IQR)	7(2.7-12.2)
Sex, female, n (%)	33(51.6)
Pre-existing disease, n (%)	5(7.8)
Cardiopathy	2(3.1)
Hematological disease	1(1.6)
Neurological disease	1(1.6)
Genetic disorders	1(1.6)
Symptoms and signs, n (%)	
Exanthema	39(60.9)
Abdominal Pain	36(56.3)
Nausea and vomits	30(46.9)
Diarrhea	24(37.5)
Dyspnea	24(37.5)
Edema of the hands and feet	20(31.3)
Conjunctivitis	15(23.4)
Cough	12(18.8)
Myalgia	12(18.8)
Irritability	9(14.1)
Skin color changes	8(12.6)
Lethargy	5(7.8)
Oliguria	5(7.8)
Tachycardia	4(6.3)
Cervical edema	4(6.3)
Lymphadenopathy	4(6.3)
Oxygen saturation less than 95%	4(6.3)
Odinophagy	3(4.7)
Dysphagia	2(3.1)
Precordial pain	2(3.1)
Mental confusion	1(1.6)
ICU admission, n (%)^a^	
Required	16 (25.0)
Not required	13(20.3)
Intensive Care Unit length of stay, days, median (IQR)	6(3.8-9.3)
Laboratorial diagnosis, n (%)	
RT-PCR	35 (54.7)
Antigen test	43(67.2)
Serology	11 (17.2)
Outcome variables^b^	
Discharge, n (%)	58(90.6)
Fatal outcome, n (%)	2(3.1)
Hospital length of stay, days, median (IQR)^c^	12(7-16)

IQR, interquartile range; RT-PCR, real-time reverse transcription-polymerase chain reaction

^a^ Data missing for 35 patients.

^b^ Data missing for 4 patients.

^c^ Data missing for 6 patients.

Five cases reported comorbidities (7.8%), such as cardiovascular disease (n =2; 3.1%), hematological disease (n =1; 1.6%), neurological disease (n =1; 1.6%), and genetic syndrome (N=1, 1.6%). Data characterizing cardiopathy were absent; however, it was described as a rheumatic fever sequelae. Electrocardiogram was available in 11 (17.2%) patients, among which, one (1.6%) patient presented with ventricular hypertrophy. Echocardiography was performed in six patients (9.3 %; n =6), wherein, two (3.1%) presented abnormalities, such as mild dilation of the left ventricle (N=1; 1.6%) and dilation of the left coronary trunk (N=1; 1.6%). Cardiac complications were reported in five (7.8%) patients: coronary aneurysm in two (3.1%), dilated cardiomyopathy in two (3.1%), and hypertrophic cardiomyopathy in one (1.6%). One of the patients with a history of cardiopathy presented with dilated cardiomyopathy.

The major clinical symptoms and signs reported were exanthema (N=39; 60.9%), abdominal pain (N=36; 56.3%), nausea and vomiting (N=30; 46.9%), diarrhea (n =24; 37.5%), dyspnea (n =24; 37.5%), hand and foot swelling (N=20; 31.3%), conjunctivitis (N=15; 23.4%), cough (N=12; 18.8%), and myalgia (N=12; 18.8%) (Table 1; Figure 2). The electrocardiogram (ECG) findings revealed severe tachycardia and significant ventricular dysfunction, which requires anticoagulation (Figure 2). One patient (n =1; 1.6%) was initially diagnosed with Kawasaki disease. A total of 16 (25.0%) children were admitted to the intensive care unit (ICU) support, among which, we identified 35 without any data (54.7%) (Table 1). Levels of C-reactive protein (75.0%), hemoglobin (51.6%), D-dimer (48.4%), lymphocytes (43.8%), LDH (45.3%), AST (42.2%), ALT (51.6%), and ferritin (48.4%) were above the reference values for a given age and gender (Table 2).

**FIGURE 2:**
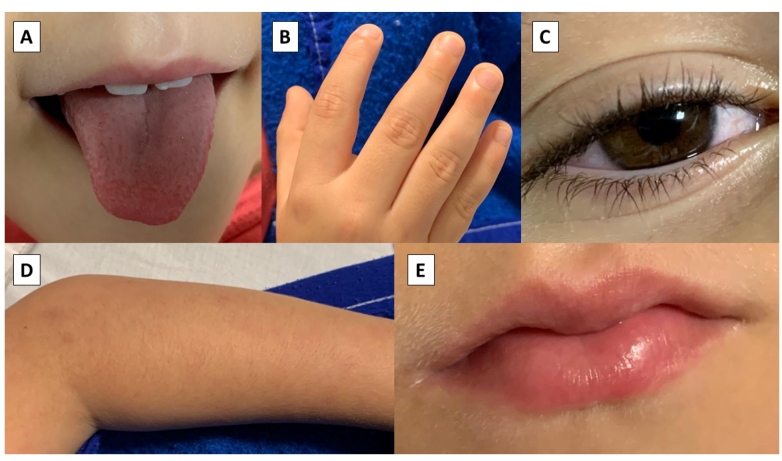
Clinical features of an 8-year-old patient with MIS-C temporally associated to COVID-19 with Kawasaki-like symptoms and signs. He also had presented tachycardia and did not evolve with skin desquamation. **A:** Strawberry tongue. **B:** Edema of the hands and feet. **C:** Bulbar conjunctival injection without exudate. **D:** Cutaneous rash. **E:** Erythema and cracking of lips. (Authors’ photo).

**TABLE 2:** Laboratorial aspects of 64 children and adolescents with MIS- C temporally associated with SARS-CoV-2 infection from Ceará, Brazil.

Laboratory	+RV	-RV	N	N/A
Hemoglobin	0	33	19	12
White cell count	17	0	37	9
lymphocytes	0	28	25	11
Platelets	23	0	31	10
C-reactive protein	48	0	9	7
D-dimer	31	0	17	15
Creatinine	14	0	38	12
Troponin	5	0	10	49
LDH	29	0	16	19
AST	27	0	28	9
ALT	33	0	21	10
Ferritin	31	0	10	22

**Legend: LDH:** lactate dehydrogenase; **AST:** aspartate aminotransferase; **ALT:** alanine aminotransferase; **+RV:** above reference values; **-RV:** below reference values; **N:** normal; **N/A:** not available.

Data regarding the treatment were available for a total of 30 patients (46.9%). Among them, 22 (73.3%) received corticosteroids, of which metiprednisolone (N=15; 68.2%) was the most common glucocorticoid prescribed. Another 23 (76.7%) patients received intravenous immunoglobulin (2 g/kg), and 8 (26.7%) patients were anticoagulated with enoxaparin (Pre-term infants: 2 mg/kg/dose; Term to 2 months: 1.5 mg/kg/dose injected subcutaneously every 12 hours; Infants ≥ 2 months and children ≤18 years: 1 mg/kg/dose injected subcutaneously every 12 hours). Moreover, seven (23.4%) children needed antibiotic therapy due to a secondary infection with ceftriaxone.

The first reported case of death was a 15-year-old female without any pre-existing chronic conditions, who succumbed to death due to sepsis after 9 days of hospitalization. The second was a 13-year-old female patient with a history of acute myeloid leukemia, declared dead after 7 days of hospitalization, without any data regarding the cause of death. Both patients were confirmed for *SARS-CoV-2* using RT-PCR. 

 These cases occur predominantly in the month of July, a month after the transmission of COVID-19 peaked. The average age of these children was 7 years (20 days-16 years). Ceará state was one of the first to confirm sustained transmission, as well as the first to show an escalation in the number of cases and deaths^8^. However, the reported deaths due to COVID-19 was confirmed by a mobile death verification service that investigated the deaths which occurred at home^9^ ^,^ ^10^.

MIS-C appears to be a clinical syndrome that shares aspects with other inflammatory conditions, wherein large amounts of cytokines cause the dysfunction of several organs and systems. Our findings were similar to other studies that reported presence of indecisive comorbidities, different from adult patients; however, it may contribute to severity and mortality^11^. This clinical picture represents a novel phenomenon that can affect previously asymptomatic children and young people with *SARS-CoV-2* infection manifesting as a hyperinflammatory syndrome with multiorgan involvement. Clinical findings are very similar to those found in cases of Kawasaki disease, KD shock syndrome, MAS, and TSS, highlighting the need for proactive surveillance and creating awareness among health professionals regarding the symptoms and the importance of an early treatment. Some differences between MIS-C and KD may be noted, such as the predominance of gastrointestinal symptoms, left ventricular systolic dysfunction, shock, and markedly elevated inflammatory biomarkers. Our report described a high incidence of gastrointestinal symptoms (abdominal pain, nausea, vomiting, and diarrhea) and elevated inflammatory biomarkers, which are commonly present in most MIS-C series. Another essential difference between MIS-C and KD is the age of onset, with MIS-C being presented at an older age as compared to KD^4^ ^,^ ^5^.

This study had several limitations. This retrospective study was conducted based on secondary data analysis. Although it represents one of the largest samples of MIS-C temporally associated with COVID-19 in the medical literature, it is still a small sample. Another major limitation is the absence of data regarding cardiac complications (myocarditis, pericarditis, and cardiac dysfunction), neurological, and renal involvement. Despite these limitations, this study provides relevant data on the pandemic settings. During the study period, there were no data regarding variant circulation such as P1 (from Manaus) and P2 (Rio de Janeiro), although genetic studies have shown that the variant N9 was born in August 2020.

All these cases occurred when interactive classes were suspended in schools, indicating that school was not the source of infection. As the reopening of schools is being planned, there has been an ongoing debate around the possibility of the children’s persisting schools being a super-disseminating site for *SARS-CoV-2* infection^12^. The present study reinforces the capability of *SARS-CoV-2* to develop severe catastrophic outcomes during the post-infectious phase. Further studies are required to fully understand the role of COVID-19 in transmission, epidemiology, clinical aspects, and severity of the disease in children. 
